# Is exposure to a climate-related disaster associated with recent experiences of intimate partner violence among women? A post hoc analysis of survey data from rural Samoa

**DOI:** 10.1136/bmjph-2024-001088

**Published:** 2025-01-16

**Authors:** Jenevieve Mannell, Pepe Tevaga, Papali’i Ene Isaako, Fa’afetai Alisi-Fesili, Louisa Apelu, Kaisarina Moananu, Taiaopo Faumuina, Lewis Sinclair, Helen Tanielu, Hattie Lowe, Laura J Brown, Andrew Copas

**Affiliations:** 1 Institute for Global Health, University College London, London, UK; 2 National University of Samoa, Apia, Samoa; 3 Samoa Victim Support Group, Apia, Samoa; 4 UNDP Spotlight Initiative, Apia, Samoa; 5 Samoa Bureau of Statistics, Apia, Samoa; 6 National University of Samoa, Toomatagi, Samoa; 7 University College London Institute for Global Health, London, UK; 8 University College London, London, UK

**Keywords:** Epidemiology, Public Health, Cross-Sectional Studies, Community Health

## Abstract

**Background:**

There is growing evidence that climate-related disasters increase rates of intimate partner violence (IPV) against women. However, there are only limited understandings of the size and nature of such associations needed to inform appropriate programming. Gaps in evidence are particularly pronounced in the Pacific—one of the regions most at risk of increased disasters from climate change.

**Methods:**

We analysed data from 450 men and 707 women collected as part of cross-sectional study of IPV experience, risk and protective factors in rural Samoan villages. Data were analysed using multivariable logistic regression models to assess associations between (1) men’s and women’s exposure to climate-related disasters and their mental health and (2) women’s exposure to climate-related disasters and their risk of IPV in the previous 12 months.

**Findings:**

Reported symptoms of depression and anxiety were associated with having experienced a disaster. Those who reported experiencing a disaster 2–3 times had 61% greater odds of reporting depression (OR 1.61; 95% CI 1.00 to 2.58) and 88% greater odds of reporting anxiety (OR 1.88; 95% CI 1.01 to 3.49), in comparison to those who reported never experiencing a disaster. Women who reported experiencing 2–3 disasters had more than twice the odds of experiencing recent IPV (adjusted OR, aOR 2.37, 95% CI 1.77 to 3.19), while those who reported experiencing 4+ disasters had over 8 times the odds (aOR 8.12; 95% CI 2.02 to 32.61).

**Interpretation:**

This is one of the first studies in the Pacific region to provide quantitative evidence of associations between exposure to climate-related events and women’s experiences of IPV. We identify a clear dose–response relationship between higher exposure to climate-related events and an increased risk of IPV for women. This points to the role of cumulative stress from experiencing repeat disasters in driving higher rates of IPV in climate-affected regions.

**WHAT IS ALREADY KNOWN ON THIS TOPIC**

**WHAT THIS STUDY ADDS**

- Survey data from rural Samoa show an association between disaster experience and poor mental health among men and women, and an association between disaster experience and intimate partner violence in the past 12 months among women.
- Multiple exposures to disasters have a dose–response relationship with women’s experience of IPV, pointing to a higher risk of violence among women exposed to multiple disasters.

**HOW THIS STUDY MIGHT AFFECT RESEARCH, PRACTICE OR POLICY**

- The study has implications for considering intimate partner violence as a potential indirect outcome of climate change in the Pacific region.

## Background

 Anthropogenic climate changes are having devastating impacts on human health globally.^1^ An increased frequency of natural hazards due to climate change, including droughts, cyclones and floods, leads to the widespread destabilisation of individuals, families and communities who are injured, killed or relocated during such events.^2^,^4^ While an increase in the severity of climate-related events is being felt all over the world, the consequences of climate change are not equally distributed and will have disproportionately large impacts on women and girls living in low-income and middle-income countries.^5^ Particularly concerning is growing evidence that the destabilisation of people’s lives and communities during and after climate-related events increases rates of intimate partner violence (IPV), as the most common form of gender-based violence against women.^5 6^

The Pacific region is currently at the sharp edge of the intersection between IPV and climate change. The region has some of the highest national prevalence rates of IPV in the world.^7^ At the same time, the Pacific is the region most vulnerable to an increase in the frequency of climate-related events.^8 9^ The geographical overlap of these two global issues is supported by recent evidence showing that countries experiencing frequent climate-related events tend to align with those that have a high prevalence of IPV.^6^ An understanding of the size and nature of associations between climate-related events and IPV is therefore critical to the development of appropriate climate change adaptation and mitigation efforts—however, to date, empirical quantitative studies on the topic remain limited.^5^

As an urgent global health concern, four scoping/systematic reviews have been completed since 2021 on the associations between climate-related events and IPV.^510^,^12^ These reviews highlight several potential mechanisms for this relationship. Severe storms, droughts and floods increase household stress and food insecurity, while at the same time reducing women’s access to support services and reifying gender inequalities in ways that increase IPV risk.^11^ Women exposed to climate-related events are more vulnerable to IPV because of its negative impact on their own mental health in ways that impact their ability to seek help and leave the violent relationship^13^ in addition to the impacts on the mental health of their husbands or partners.^14^ However, these reviews also highlight enormous gaps in the current literature. While women are disproportionately more likely to be impacted by climate change and IPV in the Pacific, the majority of the literature is from the USA and Asia, with a smaller percentage from sub-Saharan Africa. Moreover, this literature relies heavily on single natural disaster events, such as Hurricane Katrina in the USA (2006)^15^,^18^ and Typhoon Haiyan in the Philippines (2013).^19^,^21^ While such studies have helped elucidate the negative effects of such events on IPV, they fail to capture the effects of an increased frequency of severe events, which is widely predicted for the 21st century.^1^

Despite extensive international research on IPV risk factors,^22^ to our knowledge, only one peer-reviewed study has quantitatively assessed exposure to climate-related events and IPV in the Pacific region. This study from Australia^23^ compared rates of IPV against women across communities that had been affected by the 2009 bushfires in Victoria at low, medium and high levels, and associations between violence and other negative postdisaster experiences for a subgroup of individuals reporting the highest rates of violence. The results showed 4.68 higher odds of violence among women residing in bushfire-affected regions and highlighted the loss of income as a significant factor contributing to the violence. While this provides valuable evidence of connections between violence and climate-related events, it provides little evidence for how a similar disaster might impact women in the Small Island Developing States (SIDS) that account for the vast majority of countries in the Pacific, which have far less social services infrastructure and a lower baseline standard of living.^24^

The bushfire study in Australia is among others from the Pacific that have assessed the impact of climate-related events on symptoms of mental health as a key factor with a well-established evidence base for its contribution to IPV.^14 25 26^ A WHO study across 13 Pacific Island countries emphasised the high priority of addressing trauma from multiple and overlapping experiences of climate-related events, including extreme weather events, heat-related illnesses, compromised water and food, vectorborne diseases and health system deficiencies.^27^ Drawing on data from the Global Burden of Disease study, the burden of mental disorders and substance abuse in the Pacific is projected to increase by 74% by 2050, placing further strain on countries’ capacity to cope with the impacts of climate change on mental health.^28^ However, neither study quantifies the associations between increasingly poor mental health in the region and IPV as a possible outcome. Other studies on the mental health impacts of climate change in Tuvalu,^29^ Samoa,^4^ Vanuatu, Fiji^30^ and Kiribati^31^ similarly gloss over its potential implications for increased IPV.

In contrast, the growing body of literature on gender relations in the Pacific frequently mentions the increased likelihood of IPV resulting from climate change; however, this literature remains underdeveloped. A recent study conducted by UN Women in 2023 showed that people with internet access in the Pacific were more likely to search online for terms related to domestic violence immediately following a disaster^32^; however, this is only a weak indicator of actual IPV experience. Increasing evidence suggests that both men’s and women’s views towards gender and attitudes towards the use of violence against women are emphasised postdisasters.^10^ For example, a qualitative study of women’s experiences of climate change in Vanuatu highlights the daily impacts of disaster events on women’s lives, gender roles and experiences of violence. In focus groups, women described the increased pressure and stress they experienced due to land erosion, loss of crops and declining food stocks, and the authors describe how such experiences reproduce and magnify existing gender norms and the use of violence as a means of maintaining women’s adherence to well-defined social roles.^33^ Women’s gender views may also be correlated with poor mental health among women who believe that IPV is acceptable being more likely to blame themselves for the violence they experience.^34^ Other studies in the Pacific region have similarly conducted interviews and focus group discussions with women about their experiences of climate change, providing a nuanced understanding of the risks posed by climate-change events for IPV, but there is little evidence of increased rates of IPV experience or perpetration beyond individual case reports.^3^

To contribute to better evidence of direct associations between climate-related disasters and IPV in one of the world’s most-affected regions, we set out to answer the following research questions. Is past experience of a disaster from natural hazards associated with experience of IPV in the past 12 months? Are experiences of disasters from natural hazards associated with self-reported symptoms of poor mental health (ie, symptoms of depression, anxiety and harmful alcohol use)? Are observed associations between a disaster experience from natural hazards and IPV in the past 12 months sustained after adjusting for the effects of poor mental health?

Our study was conducted in rural Samoa, a Pacific country with a population of approximately 200 000 people living primarily on two large islands (Upolu and Savai’i). It is categorised as a SIDS by the United Nations and a Least Developed Country by the World Bank with a purchasing power parity for Samoans of US$6490.^35^ Following colonisation by Germany in the 20th century (1900–1914) and later by New Zealand (1914–1962), the local economy is sustained largely by remittance flows from the Samoan community in New Zealand into Samoa.^36^ Samoan history and culture are over 3500 years old, and many ancient traditions are maintained, particularly in rural areas, including the traditional Matai chiefdom structure at the village level and the organisation of social life around extended family networks called aiga potopoto.^37^

Samoa was hit by cyclones in 1990, 1991, 2004 and 2012, as well as a tsunami in 2009.^38^ Over 40% of the population is reported as having been affected by disasters due to natural hazards, with significant consequences for the economic stability of households and gross domestic product.^39^ Moreover, the majority (70%) of the population lives on customary land along the coastline, increasing their vulnerability to tropical cyclones, tsunamis and flooding.^40^ Similar to other countries in the region, Samoa has a high prevalence of IPV with 39.6% of women aged 15–49 years reporting having experienced physical, sexual and/or emotional violence from their husband or partner in their lifetime according to the 2019–2020 Demographic and Health Multiple Indicator Cluster Survey.^41^

## Methods

### Study design

Our analysis draws on data collected for a cross-sectional study of IPV experience, risk and protective factors in rural Samoan villages as part of the EVE Project (Evidence for Violence prevention in the Extreme). For the project, 10 village communities were recruited to ensure diversity in reporting cases of IPV to the Samoa Victim Support Group (SVSG)—Samoa’s largest provider of tertiary support services with a network of over 1000 village representatives, and the country’s only shelter for women and children experiencing violence. We randomly selected 200 households from each of the 10 villages based on census data. In villages with fewer than 200 households, all households were included. The sample size was calculated to assess the feasibility of a pilot intervention to reduce women’s experiences of physical and/or sexual violence in Samoa.

SVSG village representatives in each village visited selected households to collect further information (names and dates of birth) for individuals. Men and women were eligible for the survey if they were permanently residing in a village participating in the study and were between 16 and 64 years of age. Those individuals who did not meet these criteria, or who were unwilling or unable to give informed consent (due to cognitive impairment or severe mental health concerns) were ineligible. 2809 individuals were eligible for the study across the 10 villages. The list of eligible participants was used to randomly invite one woman from the first 130 households and one man from the remaining 70 households in each village to complete the survey. In villages with fewer than 200 households, a ratio of 2:3 was used to assign men’s and women’s households, respectively. An average of 130 women and 70 men were selected per village and invited by their SVSG village representatives to attend a ‘village health survey’ on a prespecified date and location. One village (representing 710 potential participants) withdrew from the study for logistical reasons (ie, urgent travel of the SVSG village representative overseas).

We minimised selection bias in this study by using National Census Data (2021) provided by the Samoa Bureau of Statistics to randomly select participating households, and then randomly select individuals from each household (average village population size 1379, range 200–4260). The comprehensiveness and reliability of the measures were improved through 3 years of qualitative work in the 10 participating villages to identify potential risk factors for IPV, and collaboratively adapting and refining survey questions and developing a theory of change for the Samoan context in partnership with village representatives.^42 43^

### Data collection

Data were collected between December 2022 and February 2023. Separate questionnaires were used to collect data for men and women. On arrival, each participant was given a randomly generated eight-digit code by an SVSG administrator to ensure the anonymity of their responses. Replacements from the same household were included in the study. Participants were matched with a trained survey enumerator of the same gender and led to a private space to conduct the survey.

Consent was requested from participants prior to completing the survey. Enumerators explained the purpose of the survey in detail and asked participants eight consent questions, recording their responses on a tablet. If the participants understood the purpose of the study and gave their consent, they were provided with the choice of completing the survey on their own or having the enumerator complete the survey on their behalf by asking questions and recording the responses. Other than consent questions, no questions were mandatory, and all participants were compensated $30 tala (~£10) for their time regardless of whether they completed the survey in full. The survey was administered using REDCap as a software interface to provide logical skip patterns and facilitate survey completion. Questionnaires took between 30 min and 1.5 hours to complete.

We followed the WHO’s ethical and safety recommendations for intervention research.^44^ Enumerators were trained using a stoplight system to identify individuals who required immediate support from a counsellor (red) versus those who could be signposted to services at a later date (orange). Counselling services were available on the day of the survey or by self-referral through an automatic form that appeared at the end of the survey for those responding yes to any of the questions marked as potentially requiring support. All participants received a paper calendar with a list of social support services and phone numbers on the back.

### Survey measures

As shown in table 1, previously validated measures were used to assess IPV prevalence and risk factors as part of the women’s survey. The men’s survey excluded questions assessing women’s experiences of IPV (DHS-MICS) and economic abuse (SEA-12).

**Table 1:** Measures used to assess IPV prevalence and risk factors

Variable	Measures
Demographics	Gender (man/woman), age, education, disability
Mental health and well-being	Self-reported depression (CES-D)^42^Generalised Anxiety Disorder (GAD-7)^43^Alcohol Use Disorder Identification Test^44^
Gender views	Gender Equity Scale^45^
Women’s experiences of violence	DHS-MICS domestic violence module^38^Scale of Economic Abuse (SEA-12)^46^
Challenging life events	Cumulative Trauma Scale^47^Household Hunger Scale^48^Childhood Experiences of Violence (EASE-PI)^49^

CES-D, Centre for Epidemiological Studies Depression Scale; DHS-MICS, Demographic and Health Survey/Multiple Indicator Cluster Survey; IPV, intimate partner violence.

Women’s experiences of physical, sexual and emotional IPV in the past 12 months were assessed using the Demographic Health Survey Multiple Indicator Cluster Survey (DHS-MICS) domestic violence module,^41^ to ensure consistency with data previously collected by the Samoa Bureau of Statistics on IPV prevalence. Women were asked questions about whether they had experienced seven acts of physical IPV: being pushed, shaken or having something thrown at them; slapped; arm twisted or hair pulled; punched; kicked, dragged or beaten up; choked or burned; threatened or attacked with a knife. They were also asked about three acts of sexual IPV: physically forced sex; physically forced sexual acts; forced with threats to perform sexual acts, and three acts of emotional IPV: humiliated; threatened with harm; insulted or made to feel bad. To assess economic violence, we reduced the Scale of Economic Abuse (SEA-12) developed by Postmus *et al*^45^ to five items, including being prohibited from getting a job or earning money; taking earnings against their will; refusing money for household expenses; excluded from financial decisions; debt built up under their name. Response options for all questions were often, sometimes, not in the past 12 months. Any positive response to the physical, sexual, emotional or economic questions was coded as having experienced IPV in the past 12 months.

Exposure to disasters due to natural hazards was assessed by a question included as part of an adapted version of the Cumulative Trauma Scale^46^: ‘*I lo’u olaga, na ou molimauina pe na ou lavea i faalavelave faalenatura e pei o mafuie, afā, asiosio poo lologa*.’ [In my life I witnessed or experienced natural events, for example, earthquake, cyclone, tornado or flood.]’ We decided to keep earthquakes as part of this question even though they are not directly related to climate change. This was intended to capture the level of destruction caused by tsunamis, which does depend on climate-related factors, including sea-level rise and the presence of natural coastal protection provided by coral reef and mangrove forests.^2^,^4^ Response options were never, once, two times, three times and many times. We generated a binary variable for never/any experience of a disaster, and a categorical variable for individuals who had never experienced a disaster, had experienced one event, had experienced two to three events or had experienced many disasters (categorised as four or more events). The emotional effect of the disaster experience was assessed by a follow-up question asking: How has this affected you? Responses were on a 6-point Likert scale ranging from extremely positively to extremely negatively.

### Depression, anxiety and harmful alcohol use

Symptoms of depression and their severity were measured using the 10-item Centre for Epidemiological Studies Depression Scale (CES-D) scale,^47^ validated in Papua New Guinea.^48^ A score was created from a sum of all 10 items (range 0–30, α=0.9), and any score equal to or above 10 was considered depressed. To assess symptoms of anxiety, we used the Generalised Anxiety Disorder Assessment (GAD-7),^49^ which includes seven items drawing on Diagnostic and Statistical Manual of Mental Disorders (DSM-IV) criteria, including feeling nervous, not being able to stop worrying, trouble sleeping, being restless and becoming easily annoyed or irritable. Response options were not at all, several days, more than half the days and nearly every day. All items were summed to create a score (range 0–21, α=0.9), and scores of 5, 10 and 15 were taken as cut-off points for mild, moderate and severe anxiety, respectively. Harmful drinking behaviours were measured using a modification of the Alcohol Use Disorders Identification Test recommended by WHO,^50^ which asks 10 questions about drinking behaviours in the past year. Values associated with a Likert scale were summed as a score (range 0–40, α=0.8), with a cut-off of 8 used to identify hazardous or harmful drinking behaviours.

### Gender views

We adapted the Gender Equal Men (GEM) scale to measure individual views on: (1) gender roles/norms and (2) acceptance of violence against women, following a systematic review of related measures, which suggested these categories were those most often associated with male perpetration of violence.^51^ The GEM scale has been adapted for many countries, while the original tool consisted of 24 questions with 2 subscales: inequitable and equitable views. Following extensive qualitative work as part of the EVE Project to adapt the inequitable subscale for the Samoan context, eight items were maintained from the original tool across two domains (acceptance of violence against women and views on gender roles) and one new item was added (‘I believe it is god’s will that a man is the head of the family’). Scores were summed separately for the two domains, with mean scores combined to create a GEM final score (range 0–18, gender roles α=0.9, acceptance of violence against women and girls α=0.7). We then created a categorical variable using scores of 4.5, 9.0, 14.0 and 18.0 as cut-off points for ‘mostly supportive’, ‘somewhat supportive’, ‘somewhat unsupportive’ and ‘mostly unsupportive’, respectively.

### Poverty

Three items were included from the Household Hunger Scale^52^ as a proxy for poverty, which have relevant factor loadings from a similar study in South Africa.^53^ These items asked how often in the past month there was no food to eat in the household; how often a member of the household had gone to sleep hungry and how often a member of the household had not eaten for a whole day and night because of no food. Response categories were never, rarely, sometimes and often. A categorical variable was created for individuals who had not experienced any of the items in the past month (none), those who rarely experienced at least one item (mild) and those who had had multiple or more severe experiences (moderate/severe).

### Childhood abuse

To assess childhood physical abuse, we included seven items from the Pacific Islands Families study, which draws on the Exposure to Abusive and Supportive Environments Parenting Inventory scale.^54^ We have drawn on these questions to provide comparability with this larger longitudinal dataset. This includes seven items about whether your parents ever: threw things at you, pulled your hair, broke or smashed objects near you when angry with you, pushed grabbed or shoved you, hit you, hit you with objects and beat you up. Response categories include never, rarely, sometimes, often, very often. Any positive response to one of the seven items was coded as having experienced child abuse.

### Patient and public involvement

A key objective of the EVE Project was to develop a locally appropriate tool for measuring the prevalence, risk and protective factors of violence against women in Samoa. As part of this objective, the project has worked in close partnership with the 10 villages involved in this study to develop the survey questionnaire and identify contextually relevant metrics. The results of this study have also been shared with these same villages and the results of these conversations have been used in our interpretation.

### Analysis

All analyses were completed in STATA SE V.17. We accounted for the sample design by calculating a proportional weight to reflect the distribution of men and women across participating villages and applied the weight to the dataset by using the svyset command in STATA.

First, we ran bivariate analyses of sociodemographic and baseline characteristics of the sample population by disaster experience as a binary variable. We report percentages and 95% CIs, or mean and 95% CI as appropriate. P values are based on survey-adapted χ^2^ tests for categorical variables and t-tests for continuous variables. Second, we examined associations between disaster experience and three mental health variables, including symptoms of depression, symptoms of anxiety and harmful alcohol use, to evaluate mental health as a potential causal pathway between disaster experience and IPV drawing on relevant literature.^23 55^ Third, we constructed multivariable logistic regression models to evaluate whether estimates of the association between women’s exposure to a disaster and their risk of IPV in the previous 12-month period persisted after adjusting for poor mental health. Symptoms of poor mental health were selected for model adjustment because of the substantial literature showing their association with IPV experience in different contexts and, therefore, their role as a potential confounder.^14 25^ Sociodemographic variables that might be acting as confounders of the relationship between disaster events and IPV were also included in all regression models. These included those that statistically confounded the association (ie, showed significant p values in the bivariate analysis) and those that have strong evidence supporting their role as covariates in similar studies. We report adjusted ORs (aORs), 95% CIs and p values for all adjusted models. There was very little missing data, although this varied across categories and is reported in relevant tables.

## Results

A total of 1169 people completed the survey (450 men and 707 women), of which 76% were on the original invite list and 24% were replacements. All respondents were from rural villages. 97% of the participants completed the survey on the tablet themselves without assistance from the enumerator. As table 2 shows, participants ranged in age from 16 to 70 years old, and nearly three-quarters of participants had completed secondary education (73.0%). There was evidence of food insecurity in the population surveyed with 20.8% reporting mild food insecurity and 7.1% reporting moderate to severe food insecurity. 29% of participants met the cut-off measure for being depressed, while rates of anxiety and harmful alcohol use were lower at 6.8% and 6.6%, respectively. 49% reported experiencing child abuse.

**Table 2:** Demographic and other baseline characteristics, associations with disaster experience

	N (%)	Missing (%)	Mean (SD)	Disaster N	Disaster % (95% CI)	P value (χ^2^ test)
Total	1169			312	26.9 (24.3 to 29.4)	
**Gender**		12 (1.0)				0.053
Men	450 (38.9)			144	32.3 (25.9 to 39.33)	
Women	707 (61.1)			166	23.6 (18.4 to 29.8)	
**Age**		0 (0.0)	61.1 (0.488)			**0**.**003**
16–19	157 (13.4)			36	23.8 (16.7 to 32.6)	
20–29	257 (21.9)			47	19.7 (13.5 to 28.0)	
30–39	232 (19.9)			63	29.3 (22.6 to 36.9)	
40–49	204 (17.5)			57	28.3 (21.6 to 36.1)	
50–59	185 (15.8)			69	38.7 (29.6 to 48.7)	
60+	73 (6.6)			21	29.0 (23.2 to 32.8)	
**Education**		30 (2.6)				**0**.**027**
Up to primary	124 (11.0)			48	40.8 (30.2 to 52.3)	
Secondary	823 (73.0)			213	26.7 (22.1 to 31.9)	
Higher education	180 (16.0)			41	25.1 (16.7 to 35.9)	
**Food insecurity**		17 (1.4)				**<0**.**001**
None	843 (72.1)			201	25.1 (21.1 to 29.6)	
Mild	243 (20.8)			79	33.6 (25.9 to 42.1)	
Moderate to severe	83 (7.1)			32	40.8 (28.4 to 53.9)	
**Disability**		52 (4.4)				**0**.**006**
Any	378 (32.3)			127	34.2 (28.3 to 40.7)	
None	791 (67.7)			185	24.0 (20.1 to 30.7)	
**Gender equality views**		19 (1.6)	9.6 (0.158)			**0**.**028**
Mostly supportive	509 (43.5)			112	23.5 (18.4 to 29.4)	
Somewhat supportive	622 (53.2)			188	31.6 (27.2 to 36.3)	
Somewhat unsupportive	38 (3.3)			12	34.6 (16.7 to 58.2)	
Mostly unsupportive	0 (0)			–	–	
**Child abuse**		34 (2.9)				**<0.001**
Any	580 (49.6)			197	34.6 (29.6 to 39.9)	
None	589 (50.4)			115	21.6 (16.6 to 27.5)	
**Poor mental health**						
CES-D	342 (29.3)	71 (6.1)	6.3 (0.171)	110	32.8 (23.4 to 43.8)	0.166
GAD-7	80 (6.8)	47 (4.0)	3.4 (0.115)	27	35.3 (26.7 to 44.9)	0.079
AUDIT	77 (6.6)	4 (<0.1)	1.4 (0.116)	29	39.6 (25.5 to 55.6)	0.110

Boldface indicates statistical significance (p<0.05).

AUDIT, Alcohol Use Disorders Identification Test; CES-D, Centre for Epidemiological Studies Depression Scale; GAD-7, Generalised Anxiety Disorder Assessment-7.

A total of 312 people reported having experienced a disaster, 26.9% of participants (table 2). Disaster exposure was higher for men (32.3%) than for women (23.6%), although this was not statistically significant. The chance of exposure to a disaster was higher among individuals with lower levels of education: 40.8% of participants who had been schooled up to primary level had experienced a disaster in contrast to 26.7% of those with secondary education and 25.1% of those with higher education. Moderate to severe food insecurity was also associated with disaster experience (40.8%) in comparison to those reporting mild (33.6%) or no food insecurity (25.1%). Bivariate associations also showed a statistically significant higher risk of experiencing a disaster if participants were disabled, had experienced child abuse and held views that were unsupportive of gender equality in comparison to those who had not had these experiences.

Depression, anxiety and harmful alcohol use were reported by 29.3%, 6.8% and 6.6% of participants, respectively. For both men and women, reporting symptoms of depression or anxiety was associated with having experienced a disaster (table 3). Experiencing multiple disasters (2–3 times) was associated with a 61% increase in the odds of reporting symptoms of depression, in comparison to those who reported never experiencing a disaster (OR 1 .61). Anxiety was also significantly associated with experiencing a disaster 2–3 times (OR 1.88). However, neither depression nor anxiety was associated with experiencing a disaster 4+ times, likely due to a low level of reporting at higher levels of exposure.

**Table 3:** Percentages, unadjusted ORs and 95% CIs for self-reported mental health by disaster exposure

	Symptoms of depression (CES-D)			Symptoms of anxiety(GAD-7)			Harmful alcohol use(AUDIT)		
	N (%)	OR (95% CI)	P value	N (%)	OR (95% CI)	P value	N (%)	OR (95% CI)	P value
**Disaster experience**									
None	230 (67.2)	1 -	--	52 (5.9)	1 -	--	48 (6.7)	1 -	--
Any	110 (32.8)	1.38(0.86 to 2.27)	0.167	27 (8.3)	1.44(0.95 to 1.17)	0.081	29 (11.2)	1.77(0.86 to 3.62)	0.113
**Frequency of disaster experience**									
None	230 (67.2)	1 -	--	52 (5.9)	1 -	--	48 (6.7)	1 -	--
Once	49 (14.6)	1.31(0.70 to 2.44)	0.373	12 (7.8)	1.35(0.69 to 2.63)	0.357	14 (12.0)	1.91(0.73 to 5.01)	0.173
2–3 times	51 (15.2)	1.61(1.00 to 2.58)	**0**.**048**	14 (10.6)	1.88(1.01 to 3.49)	**0**.**046**	9 (8.4)	1.28(0.64 to 2.56)	0.457
Many times (4+)	10 (3.1)	0.98(0.39 to 2.41)	0.959	1 (2.1)	0.34(0.04 to 3.03)	0.310	6 (17.9)	3.05(0.85 to 10.89)	0.082

Boldface indicates statistical significance (p<0.05).

AUDIT, Alcohol Use Disorders Identification Test; CES-D, Centre for Epidemiological Studies Depression Scale ; GAD-7, Generalised Anxiety Disorder Assessment-7.

Among women, 24.1% reported IPV (including physical, sexual, emotional or economic IPV) in the past 12 months. Table 4 reports the results of the association between women’s exposure to disaster and their experience of IPV in the past 12 months adjusting for age, education, food insecurity, disability, previous experience of child abuse and gender views. The models suggest that after adjustments, the association between exposure to a disaster and IPV in the past 12 months remained significant. However, this association appears to be largely driven by those experiencing multiple events, as shown by the non-significant relationship of exposure to only one disaster with IPV experience in the past year. When women’s mental health is added to the model, any exposure to a disaster and more frequent exposure are both associated with IPV experience in the past year. Women who reported experiencing 2–3 disaster events had more than twice the odds of experiencing IPV in the past 12 months (aOR 2.37), while those who reported experiencing 4+ disasters had over eight times the odds (aOR 8.12).

**Table 4:** Number, percentages and ORs for women’s experiences of IPV in the past 12 months by exposure to disaster events and mental health

	Women reporting IPV N (%)	Adjusted for demographics without mental health* aOR (95% CI)	Adjusted for demographics with mental health† aOR (95% CI)
**Disaster experience**			
None	84 (19.4)	1 -	1 -
Any	53 (39.9)	**2**.**34** (**1**.**46 to 3**.74)**	**2**.**29** (**1**.**44 to 3**.64)**
**Frequency of disaster experience**			
None	84 (19.4)	1 -	1 -
Once	16 (30.2)	1.62 (0.87 to 3.03)	1.53 (0.87 to 2.69)
2–3 times	29 (43.9)	**2**.**49** (**1**.**83 to 3**.39)***	**2**.**37** (**1**.**77 to 3**.19)**
Many times (4+)	8 (57.1)	**6**.**89** (**1**.**76 to 27**.05)**	**8**.**12** (**2**.**02 to 32**.61)*
**Poor mental health**			
No depression	80 (58.4)		1 -
Depression	57 (41.6)		1.53 (0.74 to 3.17)
No anxiety	114 (21.8)		1 -
Anxiety	23 (52.3)		**2**.**10** (**1**.**02 to 4**.32)*

Boldface indicates statistical significance.

*p<0.05; **p<0.01; ***p<0.001

* Model 1 adjusted for age, education, food insecurity, disability, child abuse, gender views.

† Model 2 adjusted for age, education, food insecurity, disability, child abuse, gender views, mental health (depression, anxiety).

IPV, intimate partner violence.

## Discussion

We assessed associations between men’s and women’s experience of climate-related events and their mental health, and women’s experience of climate-related events and their experience of IPV in the past 12 months in rural Samoa. Women’s experience of multiple climate-related events is associated with an increased risk of IPV in the past 12 months. This association remains significant after adjusting for women’s poor mental health, pointing to the potential importance of her husband or partner’s mental health postdisaster as a key driver of IPV in this setting. A woman’s experience of disasters may be highly correlated with her partner’s experiences of disasters, as a potential pathway towards increasing women’s risk of experiencing IPV. These results support other studies that have shown associations between exposure to climate-related events and women’s experiences of IPV globally,^10 11 55^ and this study is one of the first to do so quantitatively in the Pacific region. Importantly, the results show a dose–response relationship between a higher exposure to events and an increased risk of IPV for women, providing evidence that the effects of climate-related events on IPV risk will be most felt by women experiencing more than one disaster, independent of their mental health. This may help to explain non-significant findings from other studies on associations between disasters and IPV by showing the importance of multiple exposures and the specific vulnerabilities that arise in areas that are the most vulnerable to climate change.^56 57^

Our findings for other risk factors for disaster exposure by men and women in rural Samoa resonate with the growing body of evidence on the risk of disasters due to natural hazards.^12 55^ Individuals with a disability are at a higher risk of exposure to a disaster^58^; and there is a disproportionate risk of both child abuse and living in a disaster-prone area for people living in poverty.^59 60^ Increasing evidence also suggests that gender views become less supportive of gender inequality postdisasters, for reasons including women being blamed by their families and the police for not fulfilling their roles in caring for husbands or children postdisaster, and men feeling inadequate when not able to provide for their families.^20 61^ Women’s gender views may also be correlated with poor mental health as women who believe that IPV is acceptable may be more likely to blame themselves for the violence they experience.^34^ However, this interpretation should be treated with caution: gender is a complex social category in the Pacific, and existing research points to the need for more evidence on how it interacts with IPV in the Samoan context.^62^

Our results also show associations between exposure to a disaster and poor mental health including depression and anxiety in Samoa. The majority of the Samoan population will remember the devastating impact of the 2009 tsunami and the 2012 cyclone, which collectively caused over US$250 million in damages to property, negatively affecting over 10% of the population.^63^ Such collective experiences have been shown to have long-term impacts on the mental health and well-being of entire populations, whether individuals are directly affected or not.^29^ While the results from our study are not definitive given the low frequency of symptoms of anxiety among our study population, they do agree with other studies of the mental health impacts of climate-related events in the Pacific.^27 64 65^ They also raise questions of whether alcohol use (which was not significantly associated with disaster exposure in our model; table 3) is a mediating risk factor for IPV in Samoa, as it is in other Pacific countries.^7^

While the study makes an important contribution to the literature on climate change and IPV, it has some limitations. The cross-sectional design limits our ability to establish temporality between disaster experience and IPV. We have used the measure of IPV in the previous 12 months to limit temporal overlap with disaster experience. However, while our analysis focuses on disaster events, individuals may also have responded affirmatively to the question posed if they had experienced more minor events. Moreover, cross-sectional data provide little information as to whether mental health is a causal factor of IPV or the reverse, and others have suggested that the relationship is bidirectional.^13^ In addition, as with most IPV measures, the measure used is self-reported and subject to reporting bias. Other self-reported measures included depression, anxiety and harmful alcohol use—none of these were clinical diagnoses and therefore open to reporting error. While we started our study with 10 villages, the village with the highest reported cases of IPV was unable to participate in the survey for logistical reasons, and this may have impacted our results. For ethical reasons, we decided not to ask men about their perpetration of violence, and this limited our ability to examine associations between poor mental health and perpetration in addition to women’s experience. Finally, the measure for disaster experience was taken from a broad scale used to assess cumulative trauma, which did not differentiate between different types of climate-related events or different experiences of the same event. As a measure from a more generalised scale, the question also did not ask about climate-related events that contribute to particular vulnerabilities in SIDS, including higher sea surface temperatures, ocean acidification and sea-level rise.^55^ Making these distinctions and teasing out the specific ways in which exposure to different and relevant climate-related events impacts IPV risk is an important area for future research. Given the cultural and linguistic diversity of the wider Pacific region, a study in Samoa may not be fully generalisable to other Pacific Islands and more research is urgently needed in other disaster-prone countries such as Tonga, Fiji and Vanuatu.^33 66^

Our results have important implications for climate change adaptation strategies in at-risk regions, highlighting the importance of prioritising those who may be most vulnerable to repeated climate-related events and conducting gender analyses that consider IPV as part of climate change strategies. The importance of integrating IPV prevention strategies into climate change adaptation efforts is well recognised,^67^ however, what these strategies should look like remains unclear. Our study highlights a need for IPV prevention programming that engages with climate change and gender specialists to ensure that we are doing more than just addressing gender norms and poor mental health as IPV risk factors—the cumulative stress and anxiety from multiple climate-related events will need to be addressed as a key component of IPV prevention now and in the future. To deliver this, organisations and government agencies focused on environmental issues will also need to work in cross-sectoral partnerships with those delivering social programmes, that is, by delivering joint disaster preparation and adaptation strategies and highlighting the risks of climate change in IPV prevention programming.

Towards this goal, more research is needed on the different types of climate-related events and the social, physical and emotional effects they have on people’s lives. In addition to more frequent cyclones and floods, climate-related disasters of particular concern include ocean acidification, biodiversity loss and the increase in infectious diseases^55^—these all need to be considered as potential events related to climate change. Moreover, we need broader understandings of the impacts different events can have on people’s lives, for instance, the loss of land due to rising sea levels has also been shown to have profound emotional effects on the mental health and well-being of Indigenous peoples in the Pacific.^64^ These nuances urgently need to be understood from a gender lens to inform better interventions and ensure we are able to mitigate the impacts on IPV for an environmentally different future.

## Data Availability

Data are available on reasonable request.
